# Subjective and objective nutritional assessment: nurses’ role and the effect of cultural differences

**DOI:** 10.1186/s12912-021-00683-3

**Published:** 2021-09-03

**Authors:** M. Gbareen, S. Barnoy, M. Theilla

**Affiliations:** 1 Meuhedet Health Services, Tel Aviv, Israel; 2 Nursing Department, Steyer School of Health Professions, Sackler Faculty of Medicine, Tel Aviv University, Health Professions building, room 310, Ramat Aviv, 6997801 Tel Aviv, Israel; 3 Nutrition Nurse, Rabin Medical Center, Beilinson Hospital, Clalit Health Services, Petah Tikva, Israel

**Keywords:** Nutritional Assessment, MNA, SANS, Cultural Differences, Social Media

## Abstract

**Background:**

Even though the nutritional assessment of chronically ill patients has a significant effect on outcomes, nurses’ time constraints in clinical encounters may make the process impractical. Also, cultural background has an effect on nutritional assessment. Patient nutritional self-assessment can ease some of the nurses’ workload. Objectives: To compare tools for subjective and objective nutritional assessment and to examine cultural differences in nutritional assessment between Jews and Arabs living in Israel.

**Methods:**

The research design was cross-sectional; data were collected from Jews and Arabs with chronic illnesses living in the community during their visit to a public health clinic. The admitting nurse performed an objective nutritional assessment (Mini Nutritional Assessment (MNA)) after the patients completed the Subjective Nutritional Assessment (SANS). The data were analyzed using descriptive statistics, Pearson’s correlation coefficients were calculated to test the relationships between the variables, and independent student t-tests were used to compare the means and differences between groups. The diagnostic accuracy of the MNA and of the SANS was determined using the area under the curve (AUC) analysis of receiver operating characteristic (ROC) curves. The agreement between the MNA and SANS measurements was estimated by a Bland Altman plot. The level of significance employed throughout the analysis was 0.05.

**Results:**

The sample was a convenience sample of 228 chronically ill patients, consisting of 121 Arabs and 107 Jews. A significant correlation was found between the subjective and objective nutritional assessments. The Bland–Altman plot demonstrated that the SANS and the MNA have a high level of agreement. Using the area under the curve (AUC) analysis of receiver operating characteristic (ROC) curves, showed an moderate diagnostic accuracy (73 % sensitivity and 30 % specificity).

**Conclusions:**

Since the patient-completed nutritional assessment requires minimal time investment by nurses and we found a significant correlation and evidence for the accuracy and agreement of the objective and subjective assessments, further studies should assess and validate the possibility of replacing the objective nutritional assessment by the subjective assessment. Cultural background has a significant effect on patients’ nutritional self-assessment; hence, culture should be considered as part of the nutritional assessment.

**Supplementary Information:**

The online version contains supplementary material available at 10.1186/s12912-021-00683-3.

## Background

Malnutrition may lead to significant complications and cause morbidity and mortality [1], which are associated with increased medical costs [2] and heightened demand for medical and social services[3]. Malnutrition is overlooked in many cases [4]. Correct diagnosis at an early stage requires nutritional assessment. Although malnutrition is a meaningful problem, it has drawn little attention in primary care. One study showed that, on average, a nutritional assessment is accomplished only after five days of hospitalization [5], which are the equivalent of the mean hospital stay [6]. The Joint Commission [7], as well as Israel’s Ministry of Health [8], advised performing nutritional screening in the first 24 h from admission to the hospital. Nevertheless, the guidelines for nutritional screening are often not followed due to nurses’ workload and lack of time [9]. In addition, since nurses care for patients 24 h a day seven days a week, they have an essential role in providing nutritional education to patients, which is part of providing quality patient care [10] .

One of the tools considered a reliable method for assessing patient malnutrition is the Mini Nutritional Assessment (MNA), which is completed by the caregiver. Completing the assessment requires about 10–20 min for each patient, considered a long time in a busy clinical environment [11]. Hence, this nutritional assessment is often not carried out, resulting in under-identification and -treatment of malnutrition.

Lately, the Subjective Nutritional Assessment (SANS) was developed and has been suggested as a possible solution for patients’ nutrition assessment [12]. This tool is a patient nutrition self-assessment, which can ensure that a nutritional assessment is carried out despite time constraints. The SANS may be able to serve as a reliable measure and replace the time consuming objective assessments. The present study was designed to examine the reliability of the SANS tool compared to the MNA for detecting nutritional disturbances among Arab and Jewish chronically ill patients living in the community.

Considering that the SANS is a subjective assessment and that studies demonstrate the well-established influence of culture on various health outcomes and on chronic illness, culture may affect the subjective nutritional assessment. Studies show that the impact of Latino culture on various nutrition outcomes and on chronic illness self-management differs from the native American measurement of food frequency questionnaire and dietary screening results [13]. These authors suggested a differential impact of culture on diet, requiring higher specificity in dietary interventions as part of health care. In another descriptive study, researchers examined the effect of culture on patients’ views of their nutrition behaviors and self-assessment. The researchers concluded that some factors, including culture, should be integrated into the nutritional assessment[14, 15]. Cultural differences can be essential factors in assessing nutritional status [16, 17]. Therefore, it is essential to examine the effect of culture on patients’ nutritional self-assessment. The purpose of this study was to understand the relationship between objective and subjective nutritional assessments and the association with culture, i.e., among Arab Israelis and Jewish Israelis living with a chronic illness in the community in Israel.

Malnutrition is a common phenomenon among chronically ill patients [18]. It is known to be prevalent among hospitalized patients [19]but is also found in the community. The estimation is that about three million people suffer from malnutrition merely in the United Kingdom, of whom 93 % live in the community .[1].

According to the Malnutrition Universal Screening Tool (MUST), 43 % of all patients admitted to Israeli hospitals were considered at nutritional risk [20]. In the community, a recent study [21] performed in Israel showed an increased prevalence of underweight people in different age groups and an increase in malnutrition among seniors for various reasons such as chronic conditions, medication, impaired mobility, etc. Therefore, revealing and identifying malnutrition could prevent complications. The elderly are often under-diagnosed and not treated for malnutrition .[1].

Nutrition assessment is considered an integral part of the nurse’s role. The Israeli Ministry of Health (2012) published guidelines specifying nutritional care for malnourished patients that demands interdisciplinary collaboration. Also, in some places around the world nurses perform initial nutrition assessments and are involved in nutritional care [22, 23] This includes four steps: (a) Nutritional assessment performed by nurses. If the patient is identified as malnourished, the nurse should proceed to the next step; (b) Construction of a nutritional care plan by the physician and dietitian; (c) Implementation of a Nutrition Therapy Program; (d) Monitoring and documenting changes in patients’ caloric intake. In this process, stages (a), (c), and (d) are carried out by nurses [10, 24]. These guidelines emphasize nurses’ important role in identifying malnutrition. Nevertheless, a recent study revealed that nurses do not consider nutritional assessment a priority [25] and are unaware that nutritional care is their responsibility [26]. Due to the crucial role of nurses in nutritional care, it is important to educate and train nurses on the subject. As demonstrated, the massive workload nurses experience is a probable barrier to carrying out a routine nutritional assessment. The SANS subjective nutritional assessment (completed by the patient) may solve this problem and promote performance of patients’ nutritional assessment.

Arab Israelis constitute about one fifth of Israel’s total population [27]. In general, Arab Israelis are more traditional and conservative than the more modern Jewish Israeli society; the two populations also differ in their cultural characteristics[28]. This study, therefore, relates to Arab Israelis and Jewish Israelis as representing two different cultures in Israel. The Arab Population Health Survey shows that within a decade, the number of Arab patients with chronic illness has doubled, and nearly one-third of those aged 21 and older live with at least one type of chronic illness [29].The cultural difference might also have an impact on various nutritional outcomes [30, 31].Therefore, the aim of this study was to identify malnutrition in chronically ill patients, and to validate the patient’s subjective nutritional assessment (SANS) by comparing it to an objective nutritional assessment (MNA), considered a gold standard nutrition assessment tool. In addition, we aimed to identify cultural differences in nutritional assessment.

## Methods

### Research design and participants

The sample was a convenience sample. The research participants were recruited at a community clinic in Israel during 2018. The required sample size was calculated power analysis by using G*Power 3.1 [32]. The sample size required for achieving a power of 0.8 (Df = 58) and α of 0.05 was 60 patients in each group, a total of 120 participants.

Altogether, 228 patients who were able to collaborate in Hebrew or Arabic and who had been diagnosed with at least one chronic illness were included in the study. The response rate was 85 %. The participants signed a consent form prior to enrolling in the study.

### Data collection and research instruments

For each patient, the clinic nurses completed the short version of the objective nutritional assessment questionnaire MNA-SF (mini nutritional assessment), comprised of six questions. The MNA-SF is a well-validated screening tool for identifying malnutrition [33]. The replies for all items were summed; scores ranged from 0 to 14. A score of 11 or more indicated a normal nutritional state, i.e., not at risk. A score of 10 or less indicated that the patient was at risk of malnutrition. In addition, we calculated the average score to compare the nutritional assessment of the two groups.

All parts of the research questionnaire took 15–25 min to complete. The time for completing the MNA-SF questionnaire alone was 8–10 min and it was completed by the nurses.

Data were collected from the patients using a self-administered questionnaire containing sections that examined the following:

- a) Socio-demographic data; including age, marital status, education, country of origin, and culture (represented by being Jewish or Arab).
- Self-Assessment Nutrition Score (SANS) (Voloshin 2018) (see Appendix 1). The SANS is a new nutritional self-assessment tool developed by our group. It has been used in two previous studies conducted in hospital settings, yielded good results, and correctly assessed the nutritional status in accordance with the MNA (unpublished data). The questionnaire included 10 statements related to subjective assessment of the respondent’s nutritional state. The questionnaire refers to general nutritional state, changes in food consumption over the past three months, mental health assessment, eating experience, changes in body weight, level of functioning, assessment of nutritional status in relation to others, amount of fluid in the body, and weight assessment. All the statements relate exclusively to patient reporting and refer to a three-month period. The patients were asked to rate their assessment of the statements on a Likert scale ranging from 0 to 9, with 0 indicating deterioration and nine indicating improvement. A high average score for the statements indicates that the subjective nutritional assessment represents a better nutritional state. The total score ranged from 0 to 9. A score above 4 indicated normal nutritional status. Scores under 4 indicated a risk of malnutrition. We calculated the average score to compare the nutritional assessment of the two groups. Cronbach’s alpha for the SANS was 0.824.

### Ethical considerations and procedure

The Helsinki committee of the HMO where data were collected approved the research and the study was also approved by the Ethics Committees of Tel-Aviv University (Israel: 04-02-10-17 HMO; 212271-18 TAU) according to the Helsinki Declaration. Two nurses working at the clinic where data collection took place disseminated the questionnaires to the patients. Before data collection, the nurses received guidance and explanations concerning the purposes of the study. All patients who arrived at the clinic and met the inclusion criteria received a clear explanation about the study in Hebrew or Arabic as appropriate and were then invited to participate in the study on a voluntary basis, and they were free to withdraw from the study at any time. The participants read and reviewed the questionnaires, including the participants’ legal rights regarding participation and confidentiality. Those who agreed signed an informed consent form and the nurse performed the objective nutritional assessment, after which the patient completed the research questionnaire, which included the objective nutrition assessment (SANS).

### Data analysis

Data were analyzed using SPSS 25 (IBM, US). Descriptive statistics were used to analyze the participants’ socio-demographic data and the variables scores. Pearson correlation coefficients were calculated to test the relationships between the variables, and independent student t-tests were used to compare the means and the differences between the groups (Arab Israelis vs. Jewish Israelis). Fisher’s z transformation was used to examine the significance of the differences between the correlations.

The diagnostic accuracy of the SANS and the MNA was estimated using the area under the curve (AUC) analysis of receiver operating characteristic (ROC) curves. In the current study, sensitivity is more essential for the purpose of diagnosing subjects with malnutrition. The ability of the two tools to recognize the numbers reflecting the true state of malnutrition is more essential than finding patients who do not have malnutrition. An AUC < 0.70, low diagnostic accuracy between the two tools and AUC in the range of 0.7 to 0.9 is considered moderate diagnostic accuracy [34]. Higher AUC is considered good. Furthermore, we performed a Bland–Altman plot. To compare the results of the two scales and to standardize the two nutritional assessment scores, a Z score transformation was performed for both scales. The X-axis is the mean of a case for both measurements and the Y-axis is the difference between the two measurements for each subject. This is done by exploring the mean difference are the predicate limits of agreement. The plot defines the intervals of agreements. To examine the variables related to the MNA a multiple linear regression (enter method) was performed. The variables entered were: SANS score, age, education, gender, culture (Arab/Jewish), and marital status. The level of significance employed throughout the analysis was 0.05.

## Results

The research population consisted of 228 patients, 117 (51.3 %) men and 111 (48.7 %) women, of whom 121 (53.1) were Arab Israelis and 107 (46.9 %) Jewish Israelis. The mean age was 59.7 (SD = 18.29). Significant differences were observed between the Arab Israelis and the Jewish Israelis in place of birth; almost all Arab Israelis were born in Israel and only a quarter of Jewish Israelis were born in Israel. Differences in education levels between Arab Israelis and Jewish Israelis were also observed. Of the Arab Israelis, 48 % had only an elementary education, compared with 13 % of Jewish Israelis. There was no significant difference between the two groups in age, sex, and chronic illness that was the reason for their visit to the clinic. The full demographic results are presented in Table 1. Concerning income, 56.2 % of Arab Israelis earned less than the average salary in Israel, compared to 29.9 % of Jewish Israelis, and about 61.7 % of Jewish Israelis reported earning an average income compared to about 36 % of Arab Israelis.

**Table 1:** Socio-demographic characteristics of the participants according to culture (*N* = 228)

Variables	Arabs	Jews	t
	M ± SD	M ± SD	
Age	58.29 ± 18.95	61.25 ± 17.48	1.22; N.S*
	N (%)	N (%)	χ2
Sex Male Female	64 (52.9) 57 (47.1)	53 (49.5) 54 (50.5)	0.26; N.S.
Marital status Married Single/divorced/widower	78 (64.4) 43 (35.6)	80 (74.7) 27 (25.3)	5.78; N.S.
Place of birth Israel Former USSR** France Other countries	120 (99.2) 1 (0.8) ------- -------	24 (22.4) 14 (13.1) 34 (31.8) 35 (32.7)	143.79; *p* < 0.001
Education: Primary/elementary school High school Academic	84 (75.7) 13 (11.7) 14 (12.6)	45 (43.0) 27 (25.2) 34 (31.8)	47.24; *p* < 0.001
The Chronic Disease that was the Cause of the visit to the clinic: Diabetes Cardiovascular disease Cancer Neurological disease Digestive system Others	49 (40.5) 41 (33.9) 4 (3.3) 8 (6.6) 15 (12.4) 4 (3.3)	33 (31.0) 36 (33.6) 5 (4.6) 5 (4.6) 15 (14.0) 13 (12.1)	10.68; N.S.

* *N.S.* Non-Significant; *USSR* Union of Soviet Socialist Republic

Regarding the nutritional assessments, the MNA-SF identified 136 (60 %) patients as at low risk and 92 (40 %) as at high risk of malnutrition. With the SANS categorization, 141 (62 %) patients were classified as at low risk and 87 (38.2 %) as at high risk of malnutrition. According to both the MNA and the SANS, Arab Israelis displayed significantly more malnutrition than Jewish Israelis. Arab Israelis had a mean MNA score of 10.4 ± 1.8, compared to a mean score of 9.0 ± 1.8 for Jewish Israelis. Similarly, according to the SANS test, Arab Israelis had a mean score of 5.3 ± 0.9, compared to 4.8 ± 0.9 for Jewish Israelis. Independent samples t-tests indicated that the nutritional status of Arab Israelis was significantly lower than that of Jewish Israelis, as found by both nutritional tests (objective and subjective), with MNA, t = 6.009; *p* < 0.001 and SANS, t = 3.66; *p* < 0.001.

**Fig. 1:**
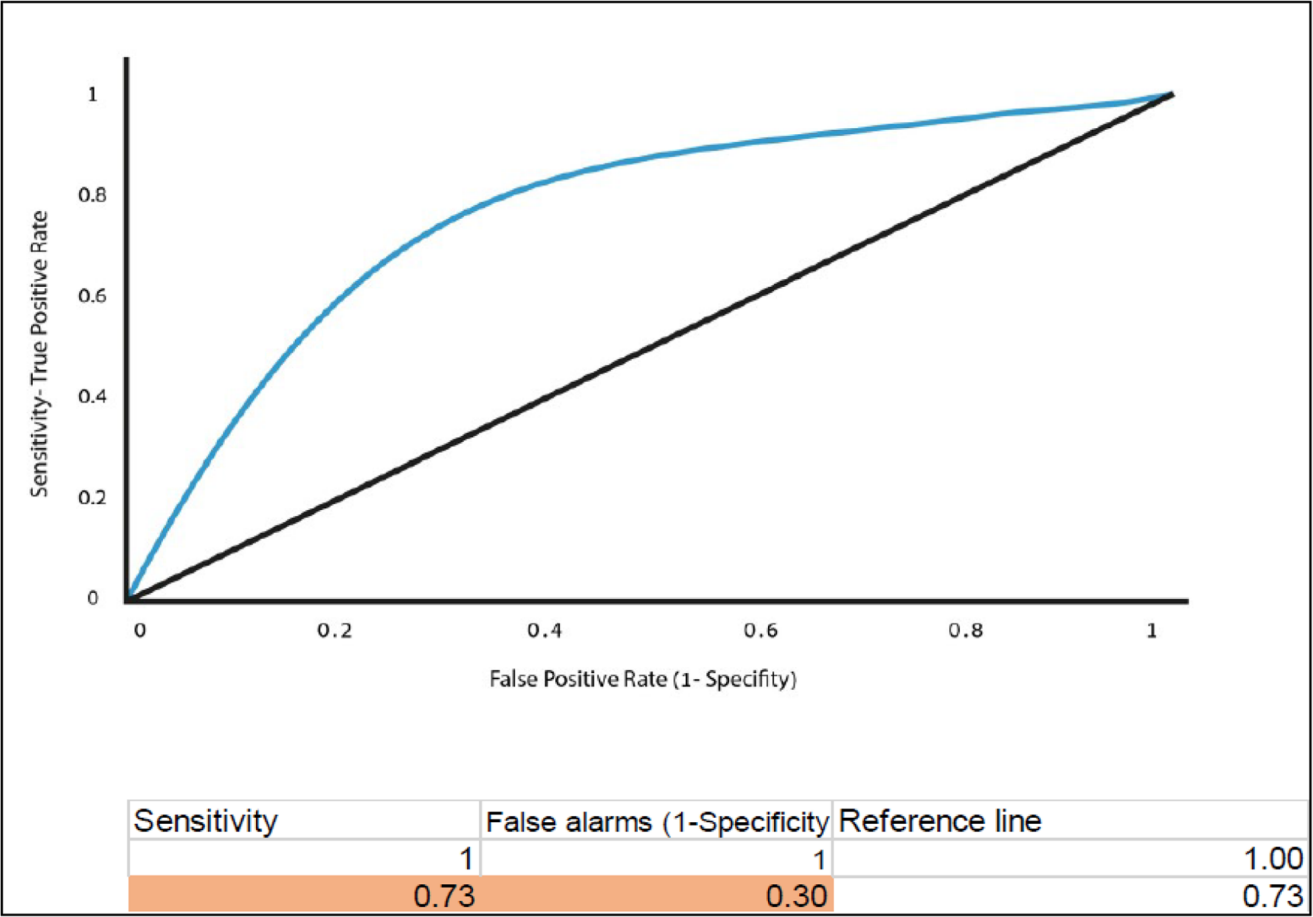
Receiver-operating characteristic (ROC) curve plot of the true positive rate (sensitivity) rate against the false positive rate (1-specificity) at SANS cut off values compared with MNA

In addition, the results and the area under curve from the ROC evaluation for the MNA, which is the gold standard for identifying patients at risk of malnutrition, compared to the SANS, showed sensitivity of 73 % and specificity of 30 %. The total area under the curve for SANS was 0.73 (*p* < 0.01) and 0.30 (see Fig. 1).

**Fig. 2:**
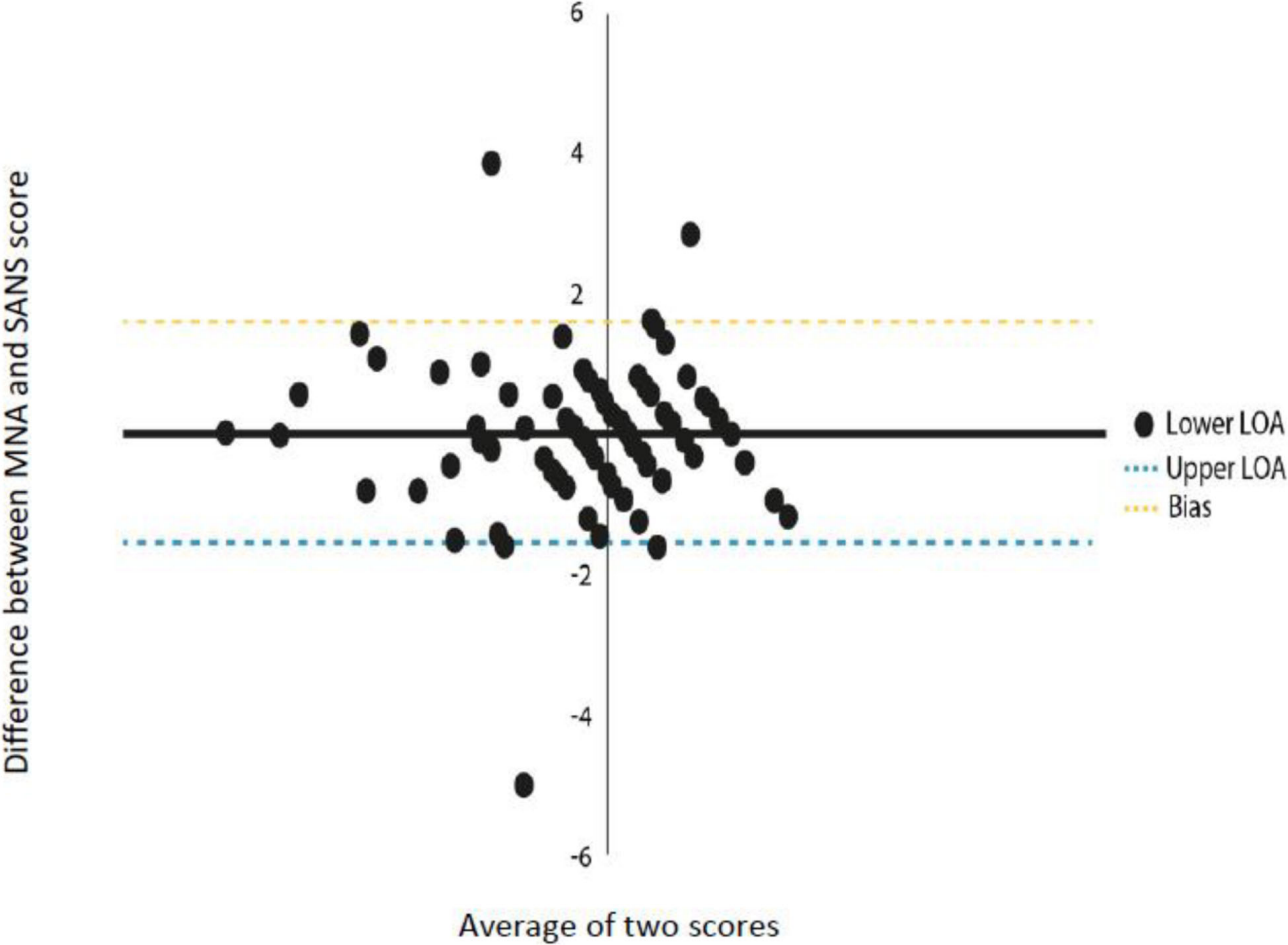
Bland-Altman plots for the two nutritional measurements. The dotted lines indicating the 95 % limits of agreement (LOA) and the straight-line indicating the mean. Dashed lines represent the regression functions of the mean of difference, upper 95 % LOA and lower 95 % LOA

We estimated the agreement between the MNA and SANS measurements by a Bland Altman plot. The measurements of the SANS and MNA were found to have a high level of agreement. The bias (mean difference of the z-scores) was 7.02*10^-7, SD ± 0.80. Of all participants, 96.49 % were found within the limits of agreement (95 % confidence interval of the mean) (see Fig. 2).

To compare the nutritional assessments of Arab Israelis and Jewish Israelis, Pearson’s correlations between the MNA and the SANS for the two groups (Arab Israelis vs. Jewish Israelis) were calculated separately. The results showed that the relationship between the two nutritional assessments in the two cultures is different, with a moderate correlation among the Jewish Israelis (*r* = 0.484, *p* < 0.001) and a high correlation among the Arab Israelis (*r* = 0.659, *p* < 0.001). Fisher’s z transformation test findings indicated that the discrepancy between the correlations for each group is significantly different (Z = -2.06, *p* < 0.05). That is, there was a higher match between the two scales among the Arab Israelis, and culture moderated the correlation between the two nutritional assessments.

In addition, to compare the normalized z-score differences in the MNA and SANS of the Arab Israelis and the Jewish Israelis, the mean for each group was calculated, revealing that the Arab Israelis’ mean difference was − 0.13 ± 0.80 versus the Jewish Israelis’ mean difference of 0.14 ± 0.94. These results show that the Arab Israelis’ subjective assessment was higher than their objective assessment, and the Jewish Israelis’ objective assessment was higher than their subjective assessment. A t-test for independent samples showed that the difference between the means of the two groups was significant (t = 2.32; *p* = 0.02).

Finally, to examine the variables related to the MNA and also find out whether there is an interaction between the SANS and culture, a multiple linear regression (enter method) was performed. The variables entered were: SANS score, age, education, gender, culture (Arab/Jewish), marital status, as there were differences between Arab and Jews participants in the SANS and MNA scores, the interaction between SANS and culture was examined. The results showed that the subjective nutritional assessment - SANS score (t = 10.15; *p* < 0.001), age (t=-326, *p* = 0.001), marital status (t = 2.93, *p* = 0.004), and culture (t = 4.94, *p *< 0.001), predicted the MNA score. That is, higher SANS scores, being younger, Jewish, and unmarried, predicted the objective nutrition evaluation, i.e., MNA scores, with the model predicting 54 % of the variance. The interaction between SANS and culture was, B = 0.388; t = 1.88; *p* = 0.078. The results are presented in Table 2.

**Table 2:** Multivariate Linear regression (Enter method) for predicting MNA score

*Variables*	B SE β t				p
SANS	1.54	0.15	0.71	10.15	< 0.001
Culture^a^	0.93	0.19	0.24	4.94	< 0.001
Age	− 0.02	0.006	− 0.18	3.26	= 0.001
Marital status^b^	− 0.77	0.26	− 0.14	2.93	= 0.004
SANS X Culture	− 0.38	0.21	− 0.12	1.88	= 0.073
**Model summary**	*p* < 0.001; R²=0.52 Adjusted R²=0.54; F = 37.16				

^a^Culture: 0 = Arab-Israeli; 1 = Jewish-Israeli

^b^Marital status: 0 = Single/divorced/widower 1 = Married

These results show a stronger association between SANS and MNA score in Arab-Israeli than Jewish-Israeli. However, the interaction did not achieve the significance level of *p* ≤ 0.05; hence these results represent a trend.

## Discussion

The present study was designed to compare the results of a subjective nutritional assessment (MNA) and those of an objective tool (SANS), with the aim of relieving part of nurses’ workload. Another purpose was to examine the effect of culture on nutritional assessment among Arab Israelis and Jewish Israelis diagnosed with a chronic illness. Overall, the results showed that both the objective and subjective nutrition assessment tools are valid and correlated with each other in moderate diagnostic accuracy level. However, cultural background had a significant effect on the patient’s nutritional self-assessment. And these findings emphasize the importance of addressing cultural aspects when performing a clinical assessment. Leininger’s theory [35] supports this conclusion. The theory seeks to understand an individual’s behavior, style of living, and social standards by his/her cultural background. Health care providers that are aware of the patient’s cultural background can prevent the deterioration of the medical condition and enhance recovery. Cultural competency is not a vague and distant aspect of nursing care, but rather a necessary part of quality health care administration [36].

The scores on the questionnaires indicated a strong correlation between the subjective (SANS) and objective (MNA) methods of nutritional assessment. Hence, the authors of the study argue that the subjective nutritional assessment questionnaire can be considered a legitimate indicator of overall nutritional status and can be used as a reliable and cost-effective measure of nutritional status. However, further studies are warranted on broad and diverse populations in order to confirm and validate this conclusion.

Many patients suffering from malnutrition are not identified during their visits to health care facilities because the available screening tools are time consuming and other urgent problems outweigh the importance of malnutrition diagnosis [37]. The fact that the self-assessing SANS tool detected malnutrition similarly to the MNA assessment, could dramatically improve the percentage of patients with malnutrition who are correctly diagnosed. This is compatible with a study that reported that a self-screening malnutrition assessment tool was as reliable when used by cancer patients as when used by dietitians [38]. The patient-led nutritional assessment requires minimal time investment by medical teams. Thus, the use of a patient nutrition self-assessment presents an attractive and efficient alternative to time consuming traditional screening procedures, although needs further validation.

The present research results of both the subjective and objective nutritional tests indicated that the nutritional state of Arab Israelis with a chronic illness was significantly worse than that of Jewish Israelis. This result is compatible with data from 2004 provided by the Israeli Center for Disease Control [39] showing that in the past 50 years there has been a significant increase in the incidence of chronic illnesses and illnesses related to Western lifestyle in the Arab Israeli population One study reported that ethnic minority groups tend to report less positive self-health than ethnic majority groups [40]. These results are consistent with the results presented here. However, we show that this trend also exists when using the objective nutrition assessment, showing that the self-reported lower nutritional status reflects the actual nutritional status.

The results presented show a significant association between culture and both MNA and SANS. In a systemic review of 92 studies [41]using an ethnographic methodology, the researchers showed that sociocultural factors are involved in the diverse scope of practice in nutrition and lead to beliefs about nutrition and knowledge of nutrition. The findings in the current study show that sociocultural factors are involved in the response diversity within the self-report of subjective and objective nutritional state. The results also show significant differences in the education and income level of Arabs compared to Jews. The level of income and education level are factors that may have a decisive effect on health status [42]. Health care organizations should be able to increase understanding and communication strategies related to nutrition and culture. Understanding these complex factors can contribute to and promote culturally tailored public health. Implementation and interventions by public health promotors will meet with success if they are culturally tailored .[43].

In a study performed almost two decades ago by Kaplan and Baron-Epel (2003), the nutrition and health status of Arab Israelis was found to be lower than that of the Jewish Israeli population. Our findings are similar and demonstrate that the deficiency in nutritional status is still present. The fact that the nutritional status has not changed over the years, shows the need to emphasize nutritional issues among Arab Israeli patients. However, Arab Israelis identified the reality of their nutritional state more accurately than Jewish-Israeli patients. This may be due to other factors such as access to health care services and trust in health care [44] and needs to be examined in further research.

The nurse’s critical thinking ability is the primary source of transcultural nursing care [45]. Nurses’ involvement in assessment and in nutritional care may reduce complications associated with malnutrition and early assessment could play a crucial role by initiation of nutritional care without delay. This was defined for the first time by Leininger [46] as “cultural competency”, which requires nurse proficiency and entails a process of communicating culturally while relating to specific health needs, such as nutrition. According to Leininger’s theory [47], health care encompasses a variety of areas affected by culture. Therefore, health care providers should continue to learn, understand, and disseminate knowledge about different values, beliefs, and ways of life. This knowledge can influence medical decisions and the quality of care.

## Conclusions

Screening and early identification of patients at nutritional risk can lead to early nutritional care. While dietitians play a significant role in the evaluation of nutritional state and recommendations, all the nutritional responsibility should be part of a multidisciplinary team. The nursing staff has an essential role in nutritional assessment and therapy. The self-administered questionnaire (SANS) was found to be highly correlated and in agreement with the nutritional assessment performed by nurses (MNA). We therefore suggest further investigation of the prospect that patient-led nutritional assessment can replace the objective nutritional assessment. This will reduce nurses’ workload and motivate nurses to be more involved in the patient’s nutritional status and care. However, further studies should assess and validate the possibility to replace the objective nutritional assessment by the subjective one. According to both nutrition assessments, the Arab Israeli population is affected by significantly more malnutrition than the Jewish-Israeli population. Cultural background has a significant effect on the patient’s self-nutritional assessment. This factor may be considered for integration in nutritional assessment for providing culturally tailored care.

## Limitations

One limitation of the study could be that all participants were treated at the same clinic. The accuracy of the subjective questionnaire could be adversely affected by recall bias when answering questions referring to the time frame, such as “Has your food intake changed in the last 3 months?“. Moreover, the fact that participants knew they would be assessed by both the subjective and objective nutritional assessments may have improved the reliability of the self-report instrument. However, the high correlation between the objective and subjective nutrition assessments points to the validity of the self-evaluation. Repetition of such a study with other participants in different settings would provide a more substantial basis for external validation of the current research findings. Low levels of income and education among Arabs could also lead to differences in responses to the questionnaire. In the future a study should be performed in similar populations to analyze the discrepancy between the two cultural groups.

## Supplementary Information

**Additional file 1:**

## Data Availability

The datasets used and analyzed in the current study are available from the corresponding author at reasonable request.

## Abbreviations

**MNA**: Mini Nutritional Assessment.
SANS: Subjective Nutritional Assessment.
**MUST**: Malnutrition Universal Screening Tool.
