# Supplementary material for: Subjective and objective nutritional assessment: nurses’ role and the effect of cultural differences
Source: BMC Nurs. 2021 Sep 3;20:157. doi: 10.1186/s12912-021-00683-3 (PMC8414867; doi:10.1186/s12912-021-00683-3)
Supplement: Supplementary file 1 — Additional file 1: [file 12912_2021_683_MOESM1_ESM.docx]

**Appendix 1 Chapter Two - Self-Assessment of Nutritional Status (SANS)**

The following questions relate to your nutritional status. You are asked to evaluate your health and nutritional status over the past three months. For each question, circle the answer that best expresses your feeling. Answers range from 1 (lowest) to 10 (highest).

1. **What is your general nutritional status today?**

| 0 | 1 | 2 | 3 | 4 | 5 | 6 | 7 | 8 | 9 |
| --- | --- | --- | --- | --- | --- | --- | --- | --- | --- |
| poor |  |  |  |  | neutral |  |  |  | excellent |

1. **In different periods of life we ​​often consume different amounts of food. Has your food intake changed in the last 3 months?**

| 0 | 1 | 2 | 3 | 4 | 5 | 6 | 7 | 8 | 9 |
| --- | --- | --- | --- | --- | --- | --- | --- | --- | --- |
| Deteriorated |  |  |  |  | Un changed |  |  |  | Improved |

1. **How do you assess your mental state and did it affect the amounts of food you consumed in the last 3 months compared to the period before?**

| 0 | 1 | 2 | 3 | 4 | 5 | 6 | 7 | 8 | 9 |
| --- | --- | --- | --- | --- | --- | --- | --- | --- | --- |
| poor |  |  |  |  | neutral |  |  |  | excellent |

1. **Sometimes people experience the eating process as one that requires effort. How would you describe your eating process experience in the past three months?**

| 0 | 1 | 2 | 3 | 4 | 5 | 6 | 7 | 8 | 9 |
| --- | --- | --- | --- | --- | --- | --- | --- | --- | --- |
| More effort |  |  |  |  | Un changed |  |  |  | Less effort |

1. **How do you estimate your weight in the last 3 months?**

| 0 | 1 | 2 | 3 | 4 | 5 | 6 | 7 | 8 | 9 |
| --- | --- | --- | --- | --- | --- | --- | --- | --- | --- |
| No need to struggle |  |  |  |  | Un changed |  |  |  | demanded more struggle |

1. **Please indicate your overall performance in the last 3 months?**

| 0 | 1 | 2 | 3 | 4 | 5 | 6 | 7 | 8 | 9 |
| --- | --- | --- | --- | --- | --- | --- | --- | --- | --- |
| Deteriorated |  |  |  |  | Un changed |  |  |  | Improved |

1. **Compared to other people your age, what is your overall nutritional status?**

| 0 | 1 | 2 | 3 | 4 | 5 | 6 | 7 | 8 | 9 |
| --- | --- | --- | --- | --- | --- | --- | --- | --- | --- |
| Much worse |  |  |  |  | Same as others |  |  |  | Much better |

1. **Please indicate changes inyour general health condition for the last 3 months?**

| 0 | 1 | 2 | 3 | 4 | 5 | 6 | 7 | 8 | 9 |
| --- | --- | --- | --- | --- | --- | --- | --- | --- | --- |
| Deteriorated |  |  |  |  | Un changed |  |  |  | Improved |

1. **How would you estimate the amount of fluid in your body?(7-9=little/no edema;4-7=mild to moderate in all areas;0-3= sever edema in most eareas)**

| 0 | 1 | 2 | 3 | 4 | 5 | 6 | 7 | 8 | 9 |
| --- | --- | --- | --- | --- | --- | --- | --- | --- | --- |
| Deteriorated |  |  |  |  | Un changed |  |  |  | Improved |

1. **How do you estimate your weight today?**

| 0 | 1 | 2 | 3 | 4 | 5 | 6 | 7 | 8 | 9 |
| --- | --- | --- | --- | --- | --- | --- | --- | --- | --- |
| Underweight |  |  |  |  | Average |  |  |  | Overweight |
